# Left bundle branch pacing is a superior pacing strategy compared to left ventricular septal pacing: insights from the pooled clinical evidence

**DOI:** 10.3389/fphys.2026.1908837

**Published:** 2026-08-24

**Authors:** Feng Li, Meng-Chao Jin, Zhi-Yuan Zhang, You Zhang, Si-Liang Peng, Hui Li

**Affiliations:** Department of Cardiology, The Second Affiliated Hospital of Soochow University, Suzhou, Jiangsu, China

**Keywords:** left bundle branch pacing, left ventricular septalpacing, heart failure, mortality, clinical outcomes

## Abstract

**Background:**

Left bundle branch pacing (LBBP) is proposed as a superior physiological pacing method to left ventricular septal pacing (LVSP). However, its comparative benefits on hard clinical outcomes remain inadequately defined.

**Methods:**

We systematically searched four databases for studies comparing LBBP with LVSP. The primary outcomes were all-cause death or heart failure hospitalization (HFH), all-cause death, HFH, or malignant ventricular arrhythmias. Subgroup analyses were performed to identify effect modifiers for the paced QRS duration and left ventricular ejection fraction (LVEF) at follow-up.

**Results:**

Fourteen studies with 1,592 patients were included. LBBP was associated with reduced risks of all-cause death/HFH (risk ratio [RR] = 0.25), all-cause death (RR = 0.30), HFH (RR = 0.26), and malignant ventricular arrhythmias (RR = 0.36). LBBP also resulted in a QRS narrowing and greater LVEF improvement. Subgroup analyses demonstrated significantly greater improvement in paced QRS duration and LVEF in the patient subgroups under 70 years of age and those with cardiac resynchronization therapy (CRT) indications.

**Conclusions:**

LBBP may be superior to LVSP, reducing mortality, heart failure, and arrhythmic events. Younger patients and those requiring CRT derive the greater benefit. These promising findings support LBBP as a preferred physiological pacing strategy, but large-scale randomized trials are needed to confirm these results.

**Clinical trial registration:**

https://www.crd.york.ac.uk/PROSPERO/view/CRD420251031432, identifier CRD420251031432.

## 1 Introduction

His bundle pacing (HBP) is regarded as the most physiological pacing modality, as it directly engages the native conduction system. However, its widespread clinical adoption is hampered by technical challenges such as low sensing amplitudes, high and unstable capture thresholds, and prolonged procedure times, which often necessitate a backup right ventricular lead (Wang et al., 2024). These limitations have spurred the development of alternative physiological pacing strategies.

Left bundle branch pacing (LBBP) has emerged as a promising alternative that combines the physiological benefits of HBP with more practical implantation characteristics (Zhang et al., 2019). By capturing the distal conduction system, LBBP achieves rapid, synchronous ventricular activation, leading to a narrow QRS complex and has demonstrated impressive clinical results, including significant improvements in left ventricular function. Nevertheless, LBBP requires precise intracardiac electrogram guidance to confirm left bundle capture and carries a potential risk of septal perforation, which may increase procedural complexity and duration (Burri and Imnadze, 2025).

In contrast, left ventricular septal pacing (LVSP) offers a comparatively simpler approach. The lead is implanted within the interventricular septum without the stringent requirement for capturing the specialized conduction system, making it a viable option when advanced equipment is unavailable or procedural efficiency is paramount (Heckman et al., 2021). Previous studies have established the feasibility and stable parameters of LVSP, showing it provides superior hemodynamics compared to traditional right ventricular pacing (Xue et al., 2025).

While both LBBP and LVSP represent significant advances over conventional pacing, it remains uncertain whether the more physiologically precise LBBP translates into superior clinical outcomes compared to the technically simpler LVSP. Current evidence is primarily derived from single-center studies with limited sample sizes, leading to inconsistent conclusions. Therefore, this meta-analysis was conducted to synthesize the available evidence and compare the clinical efficacy and safety of LBBP versus LVSP.

## 2 Methods

### 2.1 Study design

This study was systematically conducted in line with the PRISMA guidelines, as well as the preferred reporting items for reviews **Supplementary Data Sheet 4**. The study protocol was pre-registered on the official PROSPERO website (https://www.crd.york.ac.uk/PROSPERO/view/CRD420251031432).

### 2.2 Search strategy

Two reviewers (F. L and Z. Y. Z) independently searched the four databases, including PubMed, Web of Science, Cochrane Library and Embase from the establishment of the online databases up to September 1^st^, 2025. The keywords mainly included “left bundle branch pacing”, “LBBP”, “left ventricular septal pacing”, and “LVSP”. The detailed search strategy is presented in the **Supplementary Text 1**. Studies comprising the clinical outcomes between LBBP and LVSP group were enrolled. The primary outcomes mainly included all-cause death or heart failure hospitalization (HFH), all-cause death, HFH, or malignant ventricular arrhythmias. It should be noted that only studies with medium- to long-term follow-up were included in the above clinical endpoint analysis, and studies reporting only perioperative events were excluded from these analyses. The secondary outcomes included paced QRS duration, QRS narrowing, LVEF at follow-up, or delta-LVEF (calculated as follow-up LVEF minus baseline LVEF). The LBBP criteria were as follows: (1) a terminal r/R wave in lead V1 or right bundle branch block morphology in lead V1; and (2) one of the LBB capture characteristics (such as short and constant left ventricle activation time less than 80ms at high/low output in V6; recording LBB potential; or QRS transition during threshold test or programmed stimulation). The LVSP was determined according to the unipolar-tip pacing with right bundle branch block morphology in lead V1 and absence of LBB capture. A manual search was also performed to screen the potential publications. In addition, the relevant corresponding authors were contacted to acquire the missing data related outcomes in their publications.

### 2.3 Study selection

Two independent reviewers (M. C. J and Z. Y. Z) screened the titles, abstracts, and full texts to identify eligible studies. Studies were included if they were randomized controlled trials, cohort, or observational designs that directly compared LBBP with LVSP and were available as full-text articles in peer-reviewed journals. All pacing indications were considered, including atrioventricular block, cardiac resynchronization therapy (CRT), pacing-induced cardiomyopathy, and bradycardia indications. For duplicate publications, only the one with the most complete data was retained. We excluded publications such as reviews, case reports, editorials, letters, animal studies, and any articles lacking original data. A third reviewer (H. L) was consulted to adjudicate any discrepancies in the selection process.

### 2.4 Data extraction and quality assessment

A pre-designed data collection form was used by two authors (Y. Z and S. L. P) to independently acquire data from the included studies. First, general study characteristics such as publication year, first author, study design, pacing indications, sample size, and follow-up were recorded. Subsequently, patient demographics and clinical characteristics were also extracted. All controversies in this process were consulted with and resolved by a third reviewer (H. L).

The quality of the observational studies was evaluated by two independent researchers (F. L and Z. Y. Z) applying the Newcastle-Ottawa Quality Assessment Scale (NOS). This instrument assesses quality across three domains, with a total possible score of nine points. Based on the total score, studies were classified as either low quality (< 6) or moderate-to-high quality (≥ 6). Any potential disagreements in scoring were resolved by the third reviewer (H. L).

### 2.5 Statistical analysis

Continuous variables were displayed as means ± standard deviations or median with interquartile range, and categorical variables were displayed as frequencies or percentages. Relative risk (RR) and corresponding 95% confidence interval (CI) were calculated for all-cause death or HFH, all-cause death, HFH, and malignant ventricular arrhythmias. We used the weighted mean difference (WMD) and 95% confidence intervals (CI) for paced QRS duration, QRS narrowing, LVEF at follow-up, and delta-LVEF. The Stata version 16.0 (http://www.stata.com) was used for statistical analyses. *P* < 0.05 was considered statistically significant.

We utilized the I-squared (I^2^) and chi-squared test to quantify and evaluate the statistical heterogeneity of studies. If the I^2^ value was < 50% and/or *P* ≥ 0.05 for the chi-squared test, we defined that the between-study heterogeneity was not substantial, and a fixed-effect model would be performed. Otherwise, a random-effect model was performed. Sensitivity analysis was conducted by sequentially omitting one study at a time to evaluate the effect of a single study on the overall risk. Potential publication bias was also assessed using Egger’s test for outcomes with more than 10 eligible studies.

Also, subgroup analysis was conducted to screen the sources of heterogeneity, as well as the potential determinants for the clinical outcomes between LBBP and LVSP group (Li et al., 2025). Based on the study characteristics, previously reported factors and other potential factors, a total of five subgroup factors were screened, including centers (Single-center vs. Multi-center), study design (Prospectively vs. Retrospectively), pacing indications (CRT indications vs. Non-CRT indications), age (years, ≥ 70 vs. < 70), and male proportion (%, ≥ 60 vs. < 60).

## 3 Results

### 3.1 Study selection and quality assessment

A total of fourteen studies (Zhang et al., 2021; Shimeno et al., 2022; Zhou et al., 2022; Kato et al., 2023; Peng et al., 2023; Zhang et al., 2023; Chen et al., 2024; Cheng et al., 2024; Diaz et al., 2024; Rijks et al., 2024; Zhu et al., 2024; Bertini et al., 2025; Briongos-Figuero et al., 2025; He et al., 2025) are eligible for final analysis, including 1032 and 560 patients in LBBP and LVSP group, respectively. The flowchart for study selection is presented in **Supplementary Figure 1** in line with the PRISMA 2020 flow diagram (https://www.prisma-statement.org/prisma-2020-flow-diagram). The study characteristics with demographic and clinical baseline are shown in **Table 1**.

Table 1The baseline characteristics of eligible studies.

**Table:** 

First author (year)	Study design				Patients type		Sample size			Age (years) Entire cohort		Gender (male, %) Entire cohort
							LBBP		LVSP			
Briongos (2025)	Single-center, Prospectively				Consecutive patients with successful LBBAP for bradycardia pacing indication and preserved left ventricular ejection fraction were selected.		114		35	77.30±9.90		52.30
Bertini (2025)	Multi-center, Prospectively				patients receiving LBBAP between November 2023 and June 2024 were screened and prospectively enrolled.		102		53	73.30±14.30		74.80
He (2025)	Two-center, Prospectively				This prospective, 2-center observational study enrolled consecutive patients with PICM who underwent upgrading to either LBBAP or BiVP		30		10	65.80±14.14		77.50
Cheng (2024)	Single-center, Retrospectively				Pacing indications included sick sinus syndrome, atrioventricular block, and atrial fibrillation with symptomatic bradycardia.		18		22	74.68±7.30		57.50
Zhu (2024)	Two-center, Prospectively				patients with reduced left ventricular ejection fraction (LVEF <50%) undergoing CRT		68		38	62.35±12.03		64.15
Rijks (2024)	Multi-center, Prospectively				Thirty‐seven patients with a pacemaker indication for bradycardia or cardiac resynchronization therapy underwent LBBAP implantation; The indications for implantation were pace‐and‐ablate in nineteen patients (51%), CRT in seven patients (19%), AV block in seven patients (19%) and sick sinus syndrome in four patients (11%).		21		12	73.00±8.00		65.00
Diaz (2024)	Multi-center, Prospectively				All the patients had guideline approved indications for CRT: LVEF <=35% and LBBB according to Strauss criteria, or LVEF <40% with expected right ventricular (RV) pacing >40% with NYHA functional class II-IV despite guideline directed medical therapy		141		31	69.38±10.58		68.60
Chen (2024)	Multi-center, Retrospectively				CRT indications included (1) LVEF<=35% and QRS duration>=120ms or (2) LVEF 36% to 50% and expected ventricular pacing burden>=40%. Patients with HFrEF were on optimal medical therapy at least 3 months before CRT device implantation.		52		25	73.05±10.94		66.20
Zhang-2 (2023)	Single-center, Prospectively				All patients received standard medications for HF treatment for at least 3 months and conformed to the following inclusion criteria (patients with symptomatic HF and CRT indications)		15		14	68.30±9.80		48.30
Peng (2023)	Single-center, Retrospectively				Patients with traditional indications for permanent cardiac pacing; 27 patients had sinus node dysfunction, 13 had AVB, 9 had atrial fibrillation (AF) with a slow ventricular rate, 8 underwent atrioventricular node ablation, and 2 patients had indications for cardiac resynchronization therapy		46		13	71.90±12.00		35.60
Kato (2023)	Multi-center, Retrospectively				Consecutive patients with bradyarrhythmia and indication for pacemaker implantation in whom LBBAP was attempted		135		112	79.60±8.30		42.50
Shimeno (2022)	Single-center, Retrospectively				The patients admitted to our hospital for pacemaker implantation due to bradycardia		27		25	78.00±12.00		33.00
Zhou (2022)	Single-center, Retrospectively				The study cohort consisted of patients with sinus node dysfunction and AV conduction block		23		23	71.91±9.39		50.00
Zhang-1 (2021)	Single-center, Prospectively				Pacing therapy indications according to 2013 ESC guidelines on cardiac pacing		78		25	71.13±9.88		55.13

**Table:** 

First author (year)	Hypertension (%)		DM (%)		AF (%)		CHD (%)			LVEF		Follow-up
	LBBP	LVSP	LBBP	LVSP	LBBP	LVSP	LBBP	LVSP		LBBP	LVSP	
Briongos (2025)	85.90 Entire cohort		28.90 Entire cohort		40.30 Entire cohort		9.40 Entire cohort			61.8±5.2 Entire cohort		18.2±7.3M
Bertini (2025)	82.30	79.20	35.20	30.10	11.00	9.00	14.70	16.90		55.30±7.60	55.90±9.70	Only Perioperative period
He (2025)	50.00 Entire cohort		12.50 Entire cohort		NA		27.50 Entire cohort			37.41±6.85 Entire cohort		20.5±12.5M
Cheng (2024)	55.60	86.40	33.30	31.80	77.80	62.50	16.70	36.40		63.90±6.70	61.60±11.20	22.1M
Zhu (2024)	50.00	34.20	23.50	21.10	30.90	42.10	25.00	28.90		33.70±7.40	30.60±7.20	28.8±15.8M
Rijks (2024)	62.00 Entire cohort		22.00 Entire cohort		70.00 Entire cohort		27.00 Entire cohort			46.00±11.00 Entire cohort		Only Perioperative period
Diaz (2024)	77.30	67.70	46.80	35.50	46.10	61.30	36.20	35.50		25.40±8.40	25.40±8.50	399(249.5-554.8)D
Chen (2024)	67.30	76.00	38.50	60.00	50.00	56.00	67.30	84.00		35.90±8.50	33.10±7.50	307(208-508)D
Zhang-2 (2023)	NA	NA	NA	NA	13.30	28.60	26.70	28.60		32.90±5.20	31.40±5.10	17(IQR 8)M
Peng (2023)	63.00	76.90	21.70	38.50	15.20	15.40	13.00	23.10		63.10±9.40	67.00±6.20	Only Perioperative period
Kato (2023)	68.80 Entire cohort		25.90 Entire cohort		3.20 Entire cohort		4.00 Entire cohort			64.0 ± 9.8 Entire cohort		Only Perioperative period
Shimeno (2022)	NA		NA		NA		NA			NA		Only Perioperative period
Zhou (2022)	69.57	73.91	8.70	21.74	21.74	34.78	NA	NA		59.61±10.36	59.96±10.14	12M
Zhang-1 (2021)	78.00	64.00	78.00	28.00	78.00	48.00	NA	NA		57.78±14.10	53.50±16.30	6M

LBBP, Left bundle branch pacing; LVSP, Left ventricular septal pacing; DM, Diabetes mellitus; AF, Atrial fibrillation; CHD, Coronary heart disease; LVEF, Left ventricular ejection fraction; D, Days; M, Months; NA, Not available.

Among fourteen studies, seven studies (Zhang et al., 2021; Shimeno et al., 2022; Zhou et al., 2022; Peng et al., 2023; Zhang et al., 2023; Cheng et al., 2024; Briongos-Figuero et al., 2025) belong to single-center design, and the remain (Kato et al., 2023; Chen et al., 2024; Diaz et al., 2024; Rijks et al., 2024; Zhu et al., 2024; Bertini et al., 2025; He et al., 2025) belong to multi-center design. Six studies are retrospective (Shimeno et al., 2022; Zhou et al., 2022; Kato et al., 2023; Peng et al., 2023; Chen et al., 2024; Cheng et al., 2024), while others are prospective (Zhang et al., 2021; Zhang et al., 2023; Diaz et al., 2024; Rijks et al., 2024; Zhu et al., 2024; Bertini et al., 2025; Briongos-Figuero et al., 2025; He et al., 2025). The pacing indication is based on CRT criteria in five studies (Zhang et al., 2023; Chen et al., 2024; Diaz et al., 2024; Zhu et al., 2024; He et al., 2025) and on non-CRT pacing indications in the remaining nine (Zhang et al., 2021; Shimeno et al., 2022; Zhou et al., 2022; Kato et al., 2023; Peng et al., 2023; Cheng et al., 2024; Rijks et al., 2024; Bertini et al., 2025; Briongos-Figuero et al., 2025). The mean follow-up time from the nine eligible studies (Zhang et al., 2021; Zhou et al., 2022; Zhang et al., 2023; Chen et al., 2024; Cheng et al., 2024; Diaz et al., 2024; Zhu et al., 2024; Briongos-Figuero et al., 2025; He et al., 2025) ranged from 6.0 months to 28.8 months. Whereas, five studies (Shimeno et al., 2022; Kato et al., 2023; Peng et al., 2023; Rijks et al., 2024; Bertini et al., 2025) only reported the perioperative events. All fourteen eligible studies are evaluated as a moderate-to-high quality (**Supplementary Table 1**).

### 3.2 The pooled primary outcomes between LBBP and LVSP group

**Figure 1:**
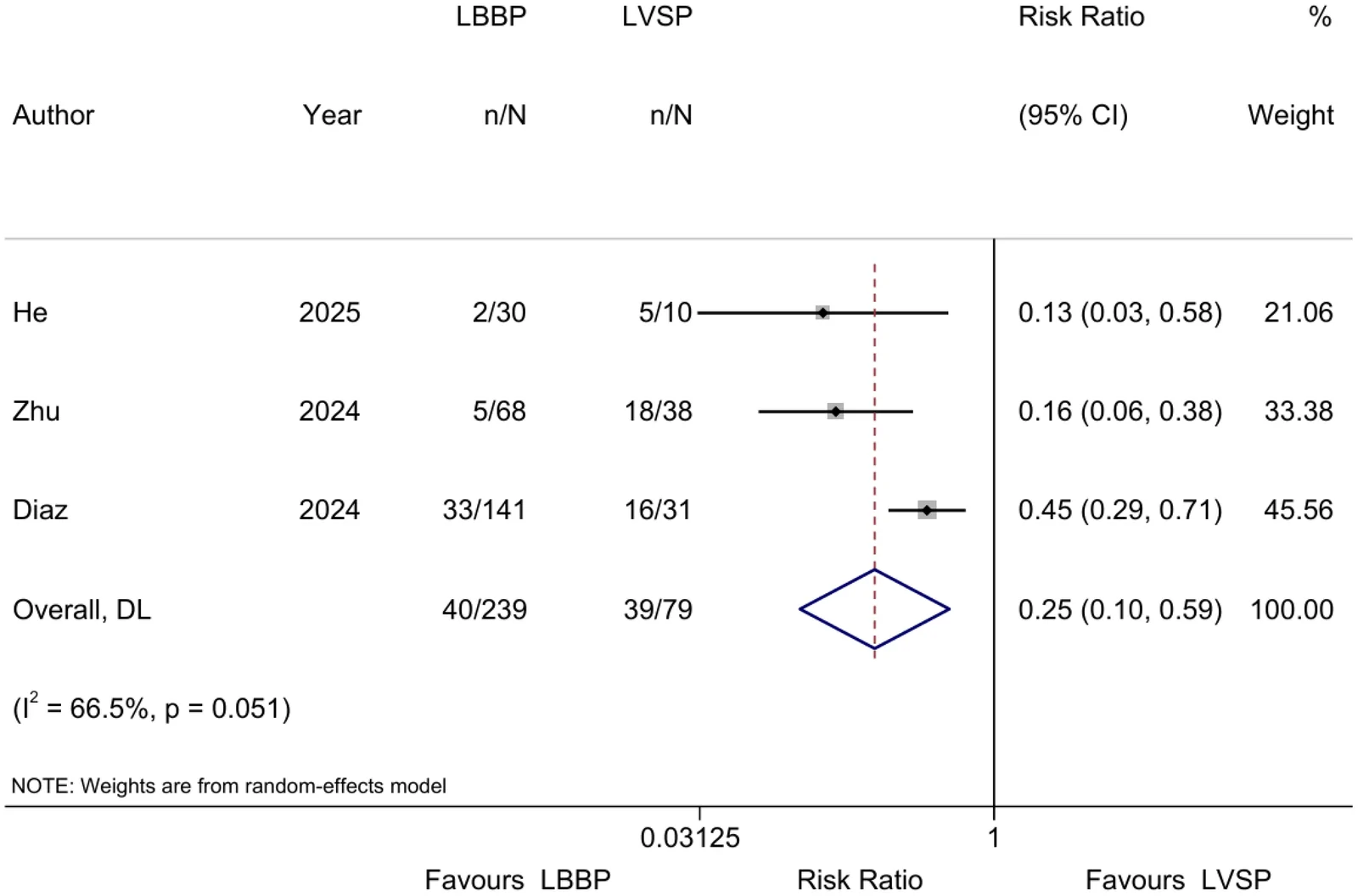
Forest plot of the rates of all-cause mortality or HFH between LBBP and LVSP group. LBBP, Left bundle branch pacing; LVSP, Left ventricular septal pacing; HFH, Heart failure hospitalization.

**Figure 2:**
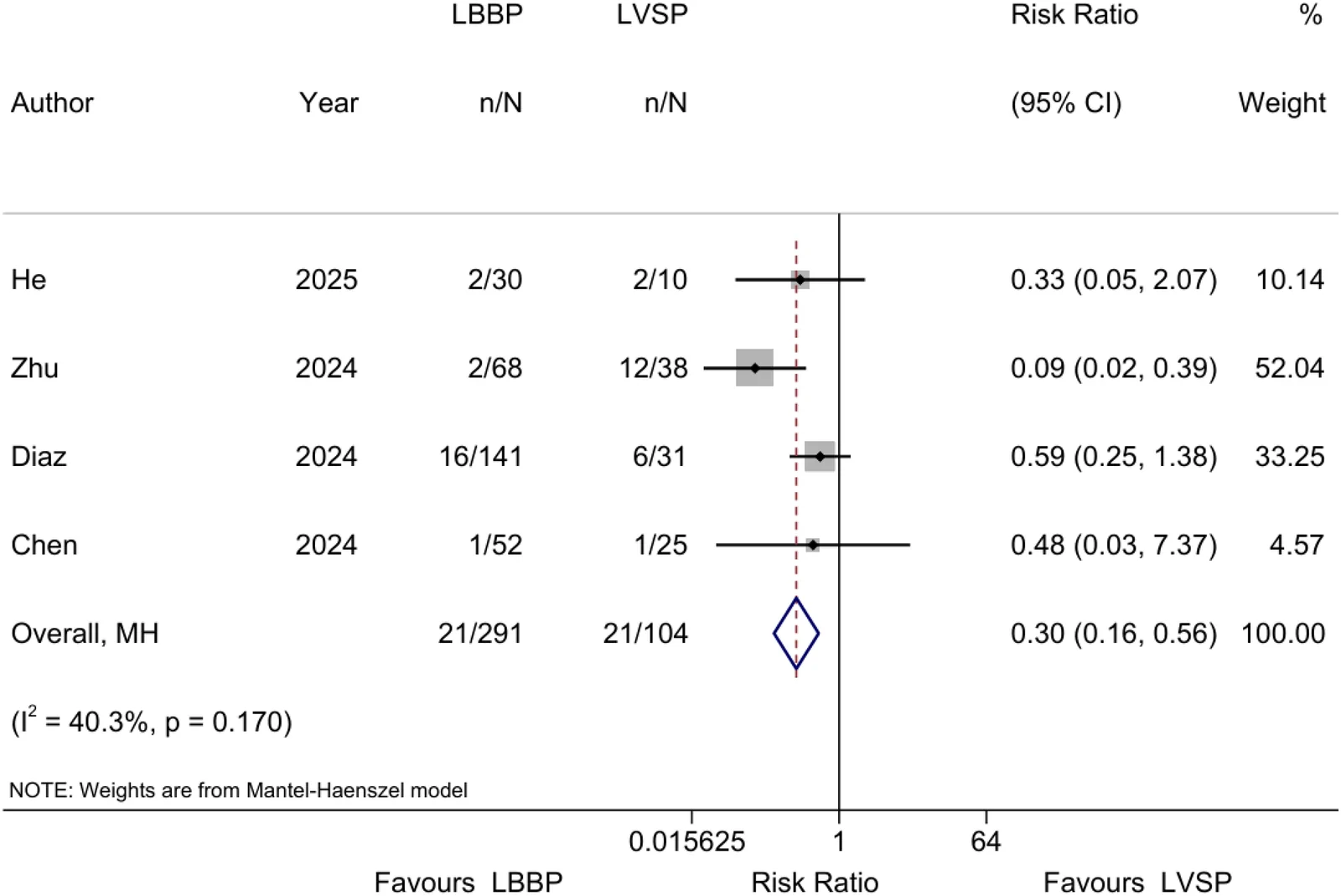
Forest plot of the rates of all-cause mortality between LBBP and LVSP group. LBBP, Left bundle branch pacing; LVSP, Left ventricular septal pacing.

**Figure 3:**
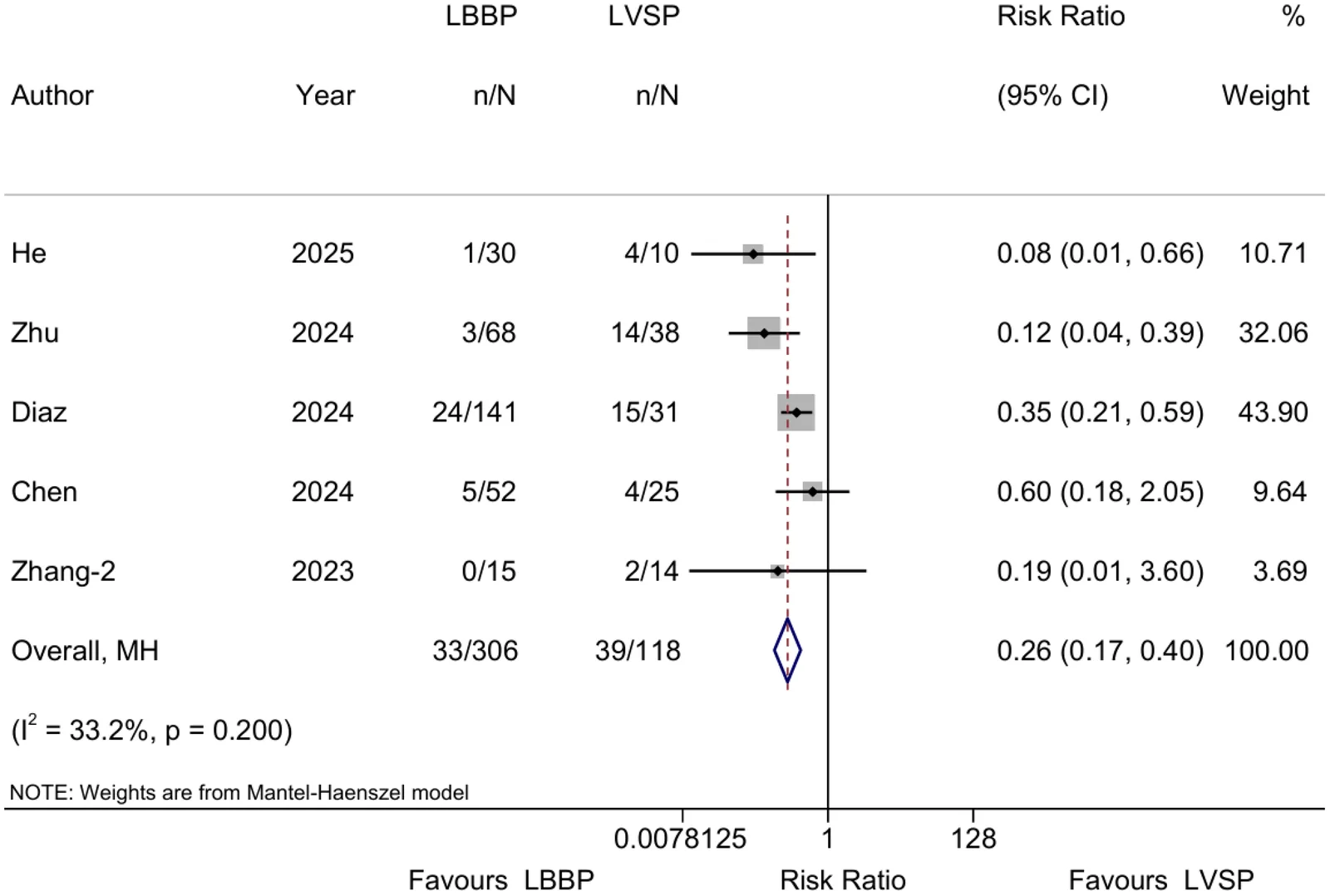
Forest plot of the rates of HFH between LBBP and LVSP group. LBBP, Left bundle branch pacing; LVSP, Left ventricular septal pacing; HFH, Heart failure hospitalization.

**Figure 4:**
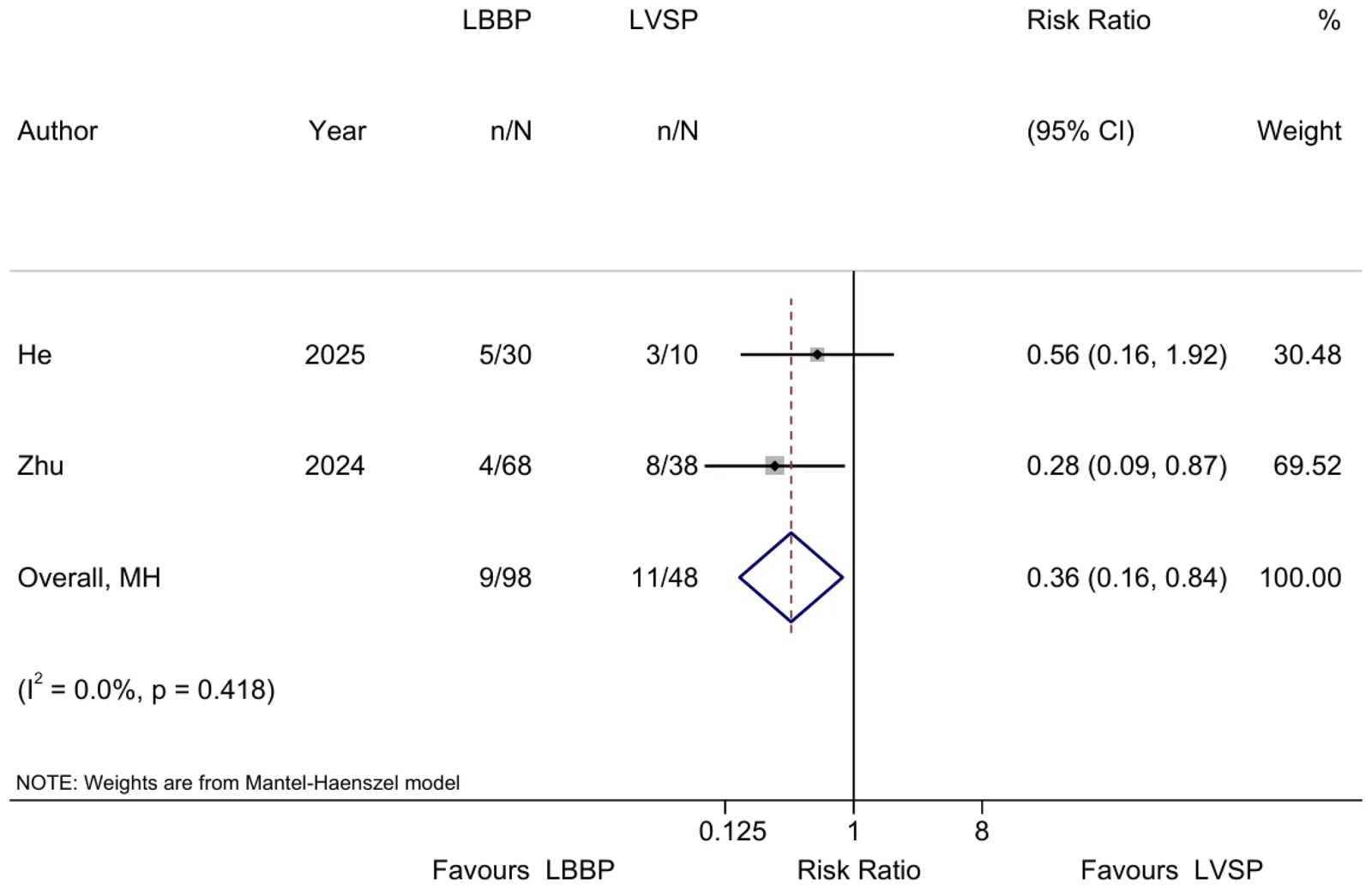
Forest plot of the rates of malignant VAs between LBBP and LVSP group. LBBP, Left bundle branch pacing; LVSP, Left ventricular septal pacing; Malignant Vas, malignant ventricular arrhythmias.

A total of three studies (Diaz et al., 2024; Zhu et al., 2024; He et al., 2025) reported the all-cause death or HFH for LBBP and LVSP group. Compared with LVSP group, LBBP group was associated with a significantly lower risk of all-cause death or HFH (RR, 0.25; 95% CI, 0.10-0.59; *P* = 0.002; I^2^ = 66.50%; **Figure 1**) via a random effect model. Four studies (Chen et al., 2024; Diaz et al., 2024; Zhu et al., 2024; He et al., 2025) reported the all-cause death for LBBP and LVSP group. Compared with LVSP group, LBBP group was associated with a significantly lower risk of all-cause death (RR, 0.30; 95% CI, 0.16-0.56; *P* < 0.001; I^2^ = 40.30%; **Figure 2**) via a fixed effect model. Five studies (Zhang et al., 2023; Chen et al., 2024; Diaz et al., 2024; Zhu et al., 2024; He et al., 2025) reported the HFH for LBBP and LVSP group. Compared with LVSP group, LBBP group was associated with a significantly lower risk of HFH (RR, 0.26; 95% CI, 0.17-0.40; *P* < 0.001; I^2^ = 33.20%; **Figure 3**) via a fixed effect model. Only two studies (Zhu et al., 2024; He et al., 2025) reported the malignant ventricular arrhythmias for LBBP and LVSP group. Compared with LVSP group, LBBP group was associated with a significantly lower risk of malignant ventricular arrhythmias (RR, 0.36; 95% CI, 0.16-0.84; *P* = 0.017; I^2^ = 0.00%; **Figure 4**) via a fixed effect model.

We also performed sensitivity analysis for the pooled risk of all-cause death or HFH, all-cause death, HFH, and malignant ventricular arrhythmias We found that no significant change in the overall combined proportion, ranging from 0.15 (95% CI, 0.07-0.32) to 0.30 (95% CI, 0.10-0.94), 0.18 (95% CI, 0.06-0.51) to 0.53 (95% CI, 0.25-1.11), 0.21 (95% CI, 0.08-0.56) to 0.35 (95% CI, 0.22-0.55), and 0.28 (95% CI, 0.09-0.87) to 0.56 (95% CI, 0.16-1.92), respectively.

### 3.3 The pooled secondary outcomes between LBBP and LVSP group

#### 3.3.1 The paced QRS duration and QRS narrowing

**Figure 5:**
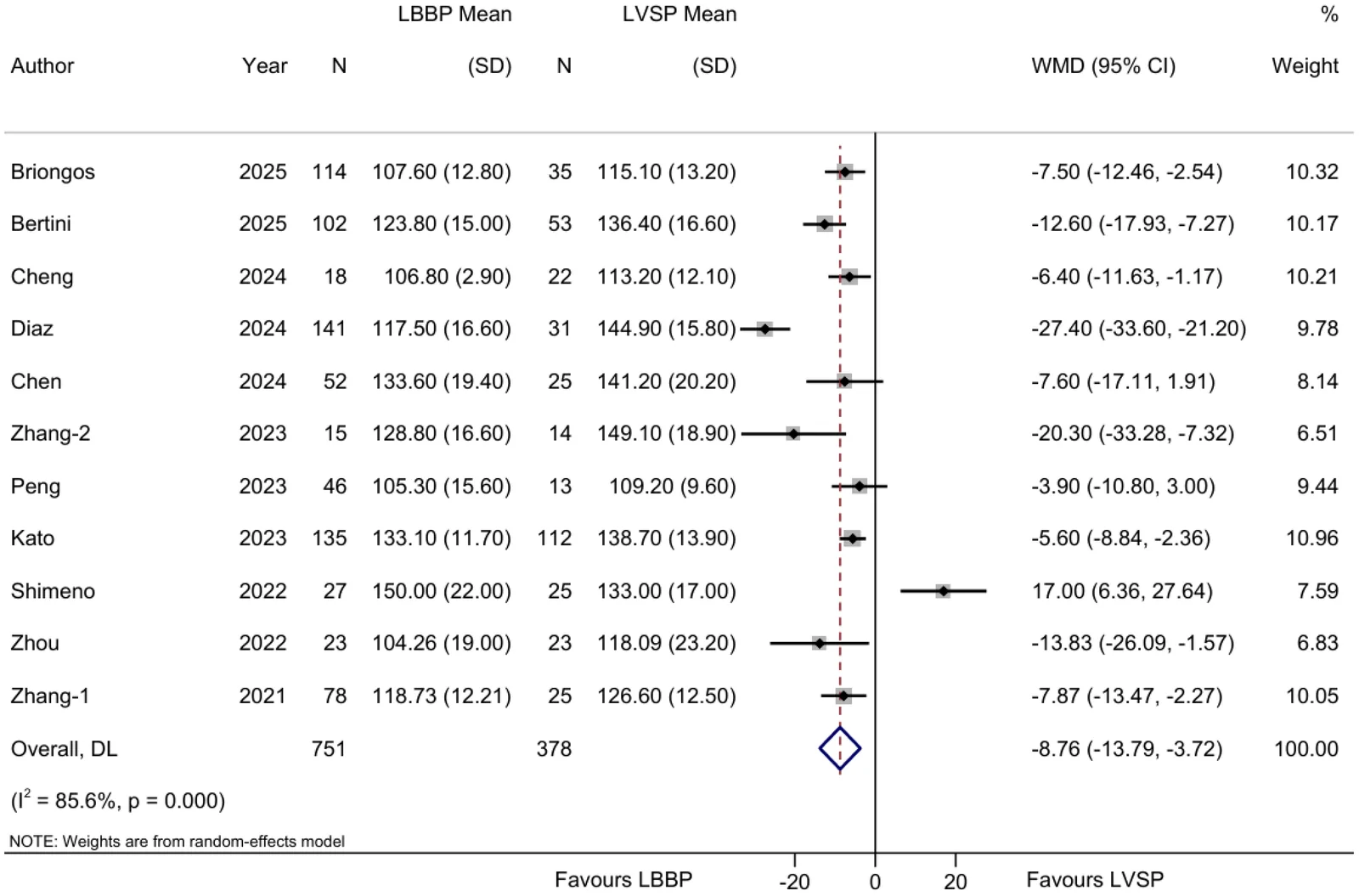
Forest plot of the paced QRS duration between LBBP and LVSP group. LBBP, Left bundle branch pacing; LVSP, Left ventricular septal pacing.

Eleven studies (Zhang et al., 2021; Shimeno et al., 2022; Zhou et al., 2022; Kato et al., 2023; Peng et al., 2023; Zhang et al., 2023; Chen et al., 2024; Cheng et al., 2024; Diaz et al., 2024; Bertini et al., 2025; Briongos-Figuero et al., 2025) reported the paced QRS duration for LBBP and LVSP group. Compared with LVSP group, LBBP group was related to a significantly narrow paced QRS duration [WMD −8.76ms; 95% CI (−13.79, -3.72); *P* = 0.001; I^2^ = 85.60%; **Figure 5**] via a random effect model. Sensitivity analysis for paced QRS duration found that WMDs ranged from -10.74ms [95% CI (-15.18, -6.30)] to -6.75ms [95% CI (-10.53, -2.96)] and the Egger’s test also showed no potential publication bias (*P* = 0.822). Notably, one study (Shimeno et al., 2022) reported a wider paced QRS duration with LBBP compared to LVSP [WMD 17.00ms; 95%CI (6.36-27.64)], representing an outlier in the opposite direction, which may reflect differences in implantation technique, lead position, or patient selection.

Meanwhile, a total of five studies (Zhou et al., 2022; Cheng et al., 2024; Rijks et al., 2024; Briongos-Figuero et al., 2025; He et al., 2025) reported the QRS narrowing for LBBP and LVSP group. Compared with LVSP group, LBBP group was associated with a higher extent of QRS narrowing [WMD 8.99ms; 95% CI (2.09, 15.90); *P* = 0.011; I^2^ = 51.20%; **Supplementary Figure 2**] via a random effect model. Sensitivity analysis for QRS narrowing found that WMDs ranged from 5.74ms [95% CI (1.51, 9.98)] to 11.22ms [95% CI (1.08, 21.37)].

Subgroup analysis identified two statistically significant intervention-covariate interactions for paced QRS duration (**Table 2**). Regarding age (*P* for interaction < 0.001), the effect was more pronounced in the <70-year subgroup [WMD −26.08 ms; 95% CI (−31.68, -20.49); *P* < 0.001] compared to the ≥70-year subgroup [WMD −6.00 ms; 95% CI (−9.71, -2.28); *P* = 0.002]. Regarding study design (*P* for interaction = 0.023), a significant effect was found in prospective studies [WMD −14.65 ms; 95% CI (−22.31, -6.99); *P* < 0.001] but not in retrospective studies [WMD −3.74 ms; 95% CI (−9.24, 1.77); *P* = 0.183].

**Table 2:** The subgroup analysis for the paced QRS duration between LBBP and LVSP.

Factors	Numbers of study	WMD	95% CI	I2 (%)	*P* value	*P* for interaction
Centers						0.197
Single-center	7	-5.82	-11.52, -0.12	76.30	0.045	
Multi-center	4	-13.31	-23.17, -3.46	92.20	0.008	
Study design						0.023
Prospectively	5	-14.65	-22.31, -6.99	86.60	0.000	
Retrospectively	6	-3.74	-9.24, 1.77	73.90	0.183	
Pacing indications						0.063
Non-CRT indications	8	-5.81	-9.84, -1.78	73.40	0.005	
CRT indications	3	-18.70	-31.67, -5.72	82.90	0.005	
Age						0.000
≥ 70 years	9	-6.00	-9.71, -2.28	69.70	0.002	
< 70 years	2	-8.76	-13.79, -3.72	0.00	0.000	
Male proportion						0.133
≥ 50%	7	-11.81	-17.52, -6.09	82.30	0.000	
< 50%	4	-2.96	-13.00, 7.09	86.30	0.564	

LBBP, Left bundle branch pacing; LVSP, Left ventricular septal pacing.

Notably, a trended towards significant intervention-covariate interaction was identified in pacing indication subgroup, including Non-CRT indication [WMD −5.81ms; 95% CI (−9.84, -1.78); *P* = 0.005] and CRT indication [WMD −18.70ms; 95% CI (−31.67, -5.72); *P* = 0.005] with *P* = 0.063 for interaction. The remaining two subgroup results (centers and male proportion subgroup) were consistent with the pooled result.

#### 3.3.2 The LVEF at follow-up and delta-LVEF

**Figure 6:**
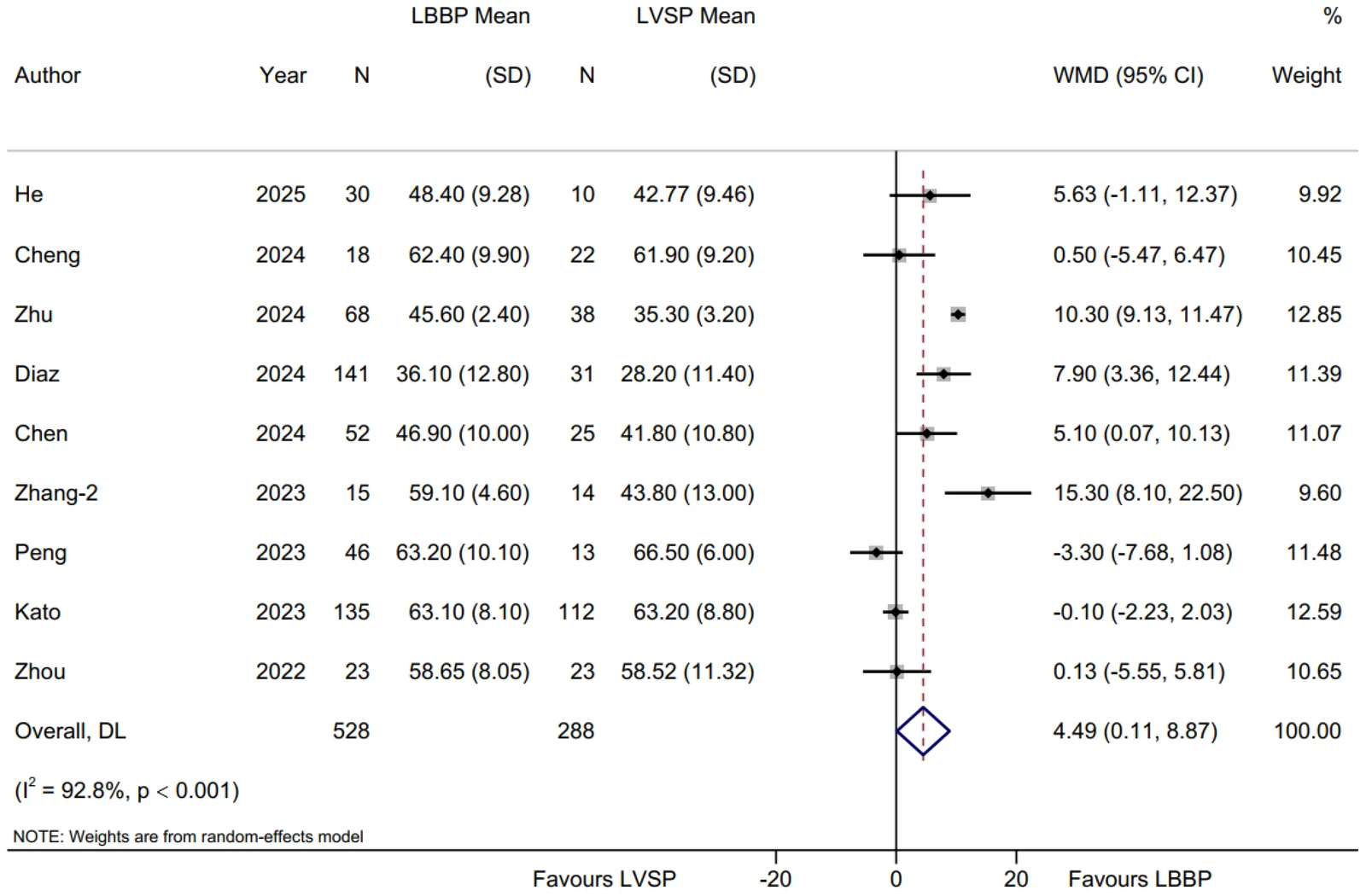
Forest plot of the LVEF at follow-up between LBBP and LVSP group. LBBP, Left bundle branch pacing; LVSP, Left ventricular septal pacing; LVEF, Left ventricular ejection fraction.

A total of nine studies (Zhou et al., 2022; Kato et al., 2023; Peng et al., 2023; Zhang et al., 2023; Chen et al., 2024; Cheng et al., 2024; Diaz et al., 2024; Zhu et al., 2024; He et al., 2025) reported the LVEF at follow-up. Compared with LVSP group, LBBP group was associated with a significant increase for the LVEF at follow-up [WMD 4.49%; 95% CI (0.11%, 8.87%); *P* = 0.044; I^2^ = 92.80%; **Figure 6**] via a random effect model. However, the extreme heterogeneity (I² = 92.80%) renders this pooled estimate difficult to interpret as a single summary statistic. The magnitude and direction of individual study estimates vary considerably, with some studies showing LBBP favoring up to 15% LVEF improvement while others show near-zero or negative differences. Therefore, this result should be interpreted with extreme caution, and the pooled estimate may not represent a meaningful clinical summary.

Sensitivity analysis for LVEF at follow-up found that WMDs ranged from 1.79% [95% CI (0.32%, 3.27%)] to 8.63% [95% CI (7.62%, 9.64%)], and the Egger’s test also showed no potential publication bias (*P* = 0.221). Three studies (Chen et al., 2024; Diaz et al., 2024; He et al., 2025) reported the delta-LVEF. LBBP group was associated with a significantly greater delta-LVEF [WMD 4.31%; 95% CI (1.71%, 6.91%); *P* = 0.001; I^2^ = 00.00%; **Supplementary Figure 3**] via a fixed effect model. Sensitivity analysis for the delta-LVEF found that WMDs ranged from 3.09% [95% CI (-0.12%, 6.29%)] to 5.63% [95% CI (2.21%, 9.06%)].

Subgroup analysis for the LVEF at follow-up between LBBP and LVSP group was conducted, and a total of three statistically significant intervention-covariate interactions were identified in study design subgroup, including prospectively subgroup [WMD 9.77%; 95% CI (7.21%, 12.33%); *P* < 0.001] and retrospectively subgroup [WMD 0.21%; 95% CI (-2.14, 2.57); *P* = 0.859] with *P* < 0.001 for interaction, in pacing indications subgroup, including Non-CRT indications [WMD -0.53%; 95% CI (-2.27%, 1.20%); *P* = 0.549] and CRT indications [WMD 8.88%; 95% CI (6.08, 11.68); *P* < 0.001] with *P* < 0.001 for interaction, as well as in age subgroup, including age ≥ 70-year subgroup [WMD -0.21%; 95% CI (-2.14%, 2.57%); *P* = 0.859] and age < 70-year subgroup [WMD 9.77%; 95% CI (7.21%, 12.33%); *P* < 0.001] with *P* < 0.001 for interaction (**Table 3**).

**Table 3:** The subgroup analysis for the followed LVEF between LBBP and LVSP.

Factors	Numbers of study	WMD	95% CI	I2 (%)	*P* value	*P* for interaction
Centers						0.518
Single-center	4	2.79	-4.37, 9.95	84.20	0.445	
Multi-center	5	5.77	0.26, 11.27	94.50	0..040	
Study design						0.000
Prospectively	4	9.77	7.21, 12.33	36.20	0.000	
Retrospectively	5	0.21	-2.14, 2.57	35.10	0.859	
Pacing indications						0.000
Non-CRT indications	4	-0.53	-2.27, 1.20	0.00	0.549	
CRT indications	5	8.88	6.08, 11.68	52.40	0.000	
Age						0.000
≥ 70 years	5	-0.21	-2.14, 2.57	35.10	0.859	
< 70 years	4	9.77	7.21, 12.33	36.20	0.000	
Male proportion						0.623
≥ 50%	6	5.37	1.45, 9.29	80.20	0.007	
<50%	3	3.22	-4.41, 10.85	89.70	0.409	

LBBP, Left bundle branch pacing; LVSP, Left ventricular septal pacing; LVEF, Left ventricular ejection fraction.

## 4 Discussion

**Figure 7:**
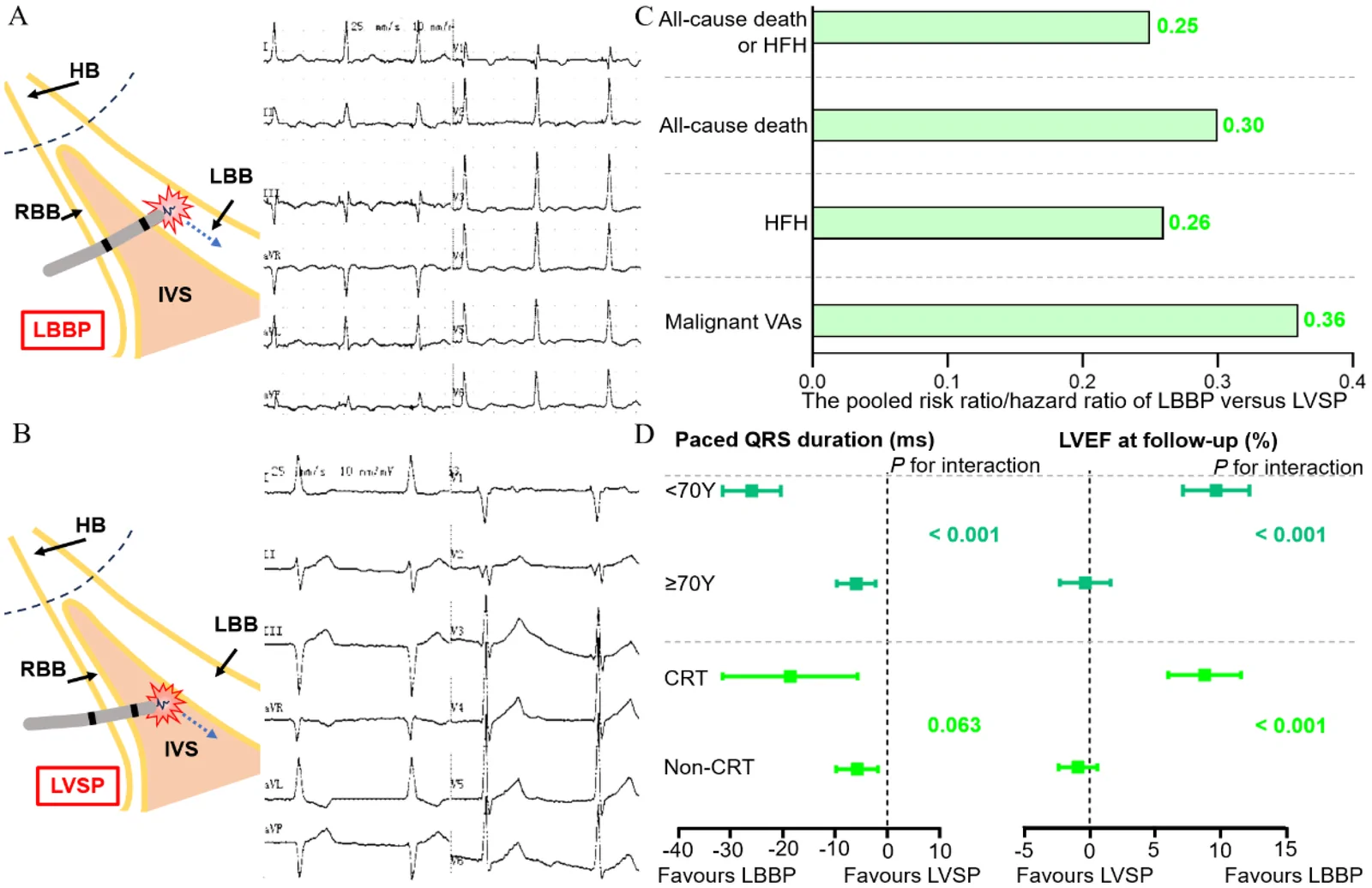
Schematic representation. **(A)** Pacing pattern diagram and related electrocardiogram for LBBP; **(B)** Pacing pattern diagram and related electrocardiogram for LVSP; **(C)** The histogram for the pooled risk ratio or hazard ratio of primary outcomes between LBBP and LVSP group; **(D)** The subgroup analysis for paced QRS duration and LVEF at follow-up with two factors (age and pacing indications, respectively). LBBP, Left bundle branch pacing; LVSP, Left ventricular septal pacing; HFH, Heart failure hospitalization; Malignant Vas, malignant ventricular arrhythmias; LVEF, Left ventricular ejection fraction.

In this study, our main findings are as follows: 1) LBBP was associated with significantly improved clinical outcomes compared to LVSP, including reduced risks of all-cause death or HFH, all-cause death, HFH, and malignant ventricular arrhythmias; 2) Two statistically significant intervention-covariates were identified for the paced QRS duration in age (*P* for interaction < 0.001) and study design (*P* for interaction = 0.023) subgroup; 3) Three statistically significant intervention-covariates were identified for the LVEF at follow-up in pacing indications, age, and study design subgroup (all *P* for interaction < 0.001). The graphic illustration is displayed in **Figure 7**.

### 4.1 Principal outcomes and physiological rationale

This meta-analysis provides the most comprehensive quantitative synthesis to date on the comparative efficacy of LBBP versus LVSP. The most notable finding is that LBBP is associated with a substantial reduction in hard clinical endpoints, including all-cause death or HFH, all-cause death, HFH, and malignant ventricular arrhythmias. This superiority is notable, with risk reductions of approximately 70-75% for the composite and individual outcomes. These findings strongly suggest that the physiological benefits of LBBP translate into meaningful long-term patient outcomes.

The electrophysiological superiority of LBBP, evidenced by the significantly narrower paced QRS duration and greater extent of QRS narrowing compared to LVSP, provides a plausible mechanism for these clinical benefits. By directly capturing the native His-Purkinje system, LBBP restores near-physiological ventricular activation, thereby promoting superior electrical synchrony (Zhu et al., 2022; Estévez et al., 2025; Gao et al., 2025; Whinnett et al., 2025). This stands in contrast to LVSP, which, while an improvement over traditional right ventricular pacing, still results in a relatively delayed and dyssynchronous activation pattern originating from the septal myocardium (Zeng et al., 2023; Curila et al., 2024). The reduction in malignant ventricular arrhythmias observed with LBBP further underscores the potential pro-arrhythmic risks of ventricular desynchrony induced by non-physiological pacing and the protective role of a more synchronous activation pattern.

### 4.2 Interpretation in the context of existing literature and subgroup analyses

Our results corroborate and significantly extend the findings from prior single-center and smaller observational studies. The pioneering work of Huang et al. (2017) and subsequent single-center cohorts, which demonstrated the feasibility and acute hemodynamic benefits of LBBP, are now validated at a pooled, meta-analytic level with robust data on hard clinical outcomes (Vijayaraman et al., 2021; Parlavecchio et al., 2023). Furthermore, our findings on QRS duration extend the findings of earlier, smaller studies (e.g., Cheng et al. (2024) on LVSP) by demonstrating a quantitatively superior effect, thereby solidifying LBBP’s position as the more physiological pacing modality. A recent study by Perea-Armijo et al. (2025) demonstrated that LBBP provides substantial benefits in patients with non-ischemic dilated cardiomyopathy and pacing-induced cardiomyopathy, with significant NT-proBNP reductions and LVEF improvements, further supporting the role of conduction system pacing in desynchrony-mediated heart failure.

The pre-specified subgroup analyses yield critical insights. The significantly greater reduction in paced QRS duration and improvement in LVEF observed in younger patients (<70 years) and those with CRT indications suggests that patients with more robust cardiac substrate and a greater pre-existing burden of desynchrony derive the maximum benefit from LBBP. This is a pivotal finding for patient selection. Similarly, the more pronounced benefits observed in prospective versus retrospective studies highlight the potential for confounding in observational data and underscore the need for rigorously designed randomized trials to confirm these effect sizes. The strong interaction showing that LVEF benefit is almost entirely driven by patients with CRT indications powerfully argues for the preferential use of LBBP in this population, where resynchronization is the primary therapeutic goal.

Moreover, some included studies enrolled CRT patients with LVEF >35%, which does not fully align with current clinical practice guideline recommendations. This may reflect real-world practice or regional variations in guideline application, and represents a potential source of heterogeneity.

### 4.3 Clinical implications and future directions

This meta-analysis supports using LBBP as a standard treatment, not just an experimental option. It should be the first-choice physiological pacing method for specific patients, including: those needing frequent ventricular pacing (Li et al., 2025), those with HF who qualify for CRT, and younger patients whose long-term heart function is a top priority. It effectively combines the physiological superiority of His-bundle pacing with the practical advantages of stable thresholds and easier implantation, while outperforming LVSP in key clinical and electrophysiological metrics. However, given the predominantly observational nature of the included studies, these recommendations should be viewed as supportive rather than definitive, and randomized controlled trials are urgently needed.

### 4.4 Study limitations

Several limitations of this study must be acknowledged. First, the included studies were predominantly observational, rendering the results susceptible to residual confounding and selection bias, despite our rigorous methodology and subgroup analyses. Second, the overall patient distribution was markedly asymmetric, including 1,032 patients in the LBBP group versus only 560 in the LVSP group. This imbalance reflects real-world clinical practice where LBBP is increasingly preferred, but may also introduce systematic bias that could influence pooled estimates. Third, there was significant heterogeneity in the pooled estimates for certain electrophysiological parameters (e.g., paced QRS duration, I^2^ = 85.6%), which, although explored through subgroup analysis, indicates variability in patient populations, implant techniques, or definitions. Fourth, the pooled LVEF estimate demonstrated extreme heterogeneity (I^2^ = 92.8%), rendering the summary statistic difficult to interpret; the benefit appears to be driven primarily by CRT-indication and younger-patient subgroups. Fifth, the mean follow-up duration across studies was variable and relatively short for a chronic device therapy; the long-term durability of LBBP leads and the sustained clinical benefits beyond two or three years require further investigation. Sixth, most included studies did not report outcomes stratified by the underlying etiology of left ventricular dysfunction (ischemic vs. non-ischemic), which may be an important effect modifier. Future research on etiological results should be conducted based on the etiological results in a hierarchical manner. Seventh, the definitions of LBBP and LVSP capture, while standardized in our protocol, might have had subtle variations in implementation across different centers. Finally, as with any aggregate data meta-analysis, we were unable to perform individual-level analyses to adjust for all potential confounders.

## 5 Conclusion

In conclusion, this meta-analysis provides evidence that left bundle branch pacing may be superior to left ventricular septal pacing in improving key clinical outcomes, including all-cause death and HFH, while also demonstrating significant advantages in electrophysiological and echocardiographic parameters. The benefits appear most pronounced in younger patients and those with conventional CRT indications. These findings support the adoption of LBBP as a preferred physiological pacing strategy when feasible, but should be considered preliminary given the limited number of studies and events, and the predominantly observational design of the included studies. Large-scale, multicenter, randomized controlled trials are warranted to confirm these findings and to establish the long-term superiority of LBBP.

## Data Availability

The original contributions presented in the study are included in the article/**Supplementary Material**. Further inquiries can be directed to the corresponding author.

## Supplementary material

Supplementary Figure 1 The flowchart for study selection.

Supplementary Figure 2 Forest plot of the QRS narrowing between LBBP and LVSP group. LBBP group was associated with a higher extent of QRS narrowing compared with LVSP. LBBP, Left bundle branch pacing; LVSP, Left ventricular septal pacing.

Supplementary Figure 3 Forest plot of the delta-LVEF between LBBP and LVSP group. LBBP group was associated with a significantly greater delta-LVEF compared with LVSP. LBBP, Left bundle branch pacing; LVSP, Left ventricular septal pacing; LVEF, Left ventricular ejection fraction.
