# Supplementary material for: Left bundle branch pacing is a superior pacing strategy compared to left ventricular septal pacing: insights from the pooled clinical evidence
Source: Front Physiol. 2026 Aug 24;17:1908837. doi: 10.3389/fphys.2026.1908837 (PMC13546746; doi:10.3389/fphys.2026.1908837)
Supplement: Supplementary file 1 [file DataSheet2.pdf]

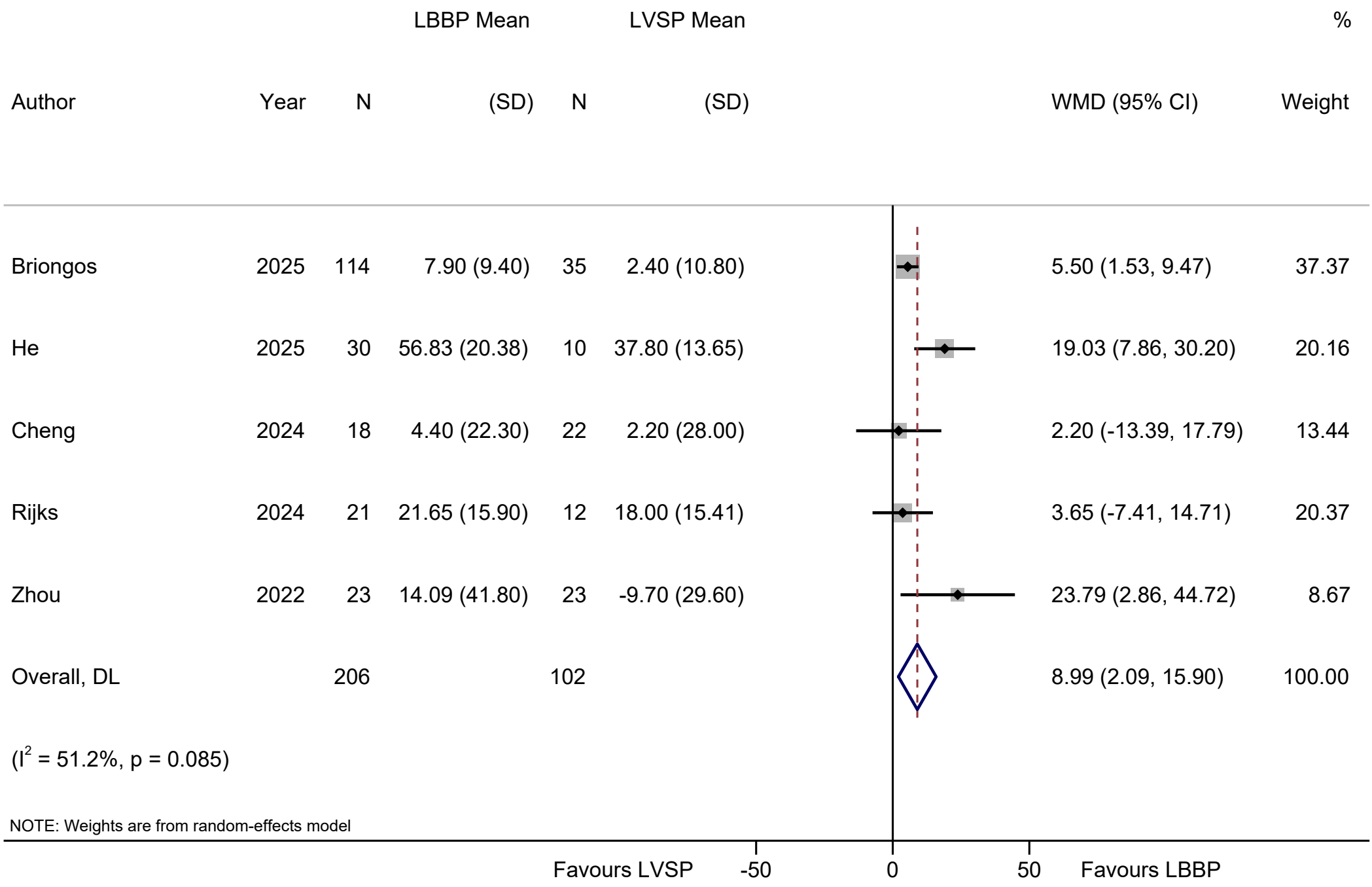

LBBP Mean
LVSP Mean
%
Author
Year
N
(SD)
N
(SD)
WMD (95% CI)
Weight
Briongos 2025 114 7.90 (9.40) 35 2.40 (10.80) 5.50 (1.53, 9.47) 37.37
He 2025 30 56.83 (20.38) 10 37.80 (13.65) 19.03 (7.86, 30.20) 20.16
Cheng 2024 18 4.40 (22.30) 22 2.20 (28.00) 2.20 (-13.39, 17.79) 13.44
Rijks 2024 21 21.65 (15.90) 12 18.00 (15.41) 3.65 (-7.41, 14.71) 20.37
Zhou 2022 23 14.09 (41.80) 23 -9.70 (29.60) 23.79 (2.86, 44.72) 8.67
Overall, DL 206 102 8.99 (2.09, 15.90) 100.00
(I^2 = 51.2%, p = 0.085)
NOTE: Weights are from random-effects model
Favours LVSP
-50
0
50
Favours LBBP
