# Supplementary material for: Left bundle branch pacing is a superior pacing strategy compared to left ventricular septal pacing: insights from the pooled clinical evidence
Source: Front Physiol. 2026 Aug 24;17:1908837. doi: 10.3389/fphys.2026.1908837 (PMC13546746; doi:10.3389/fphys.2026.1908837)
Supplement: Supplementary file 2 [file DataSheet3.pdf]

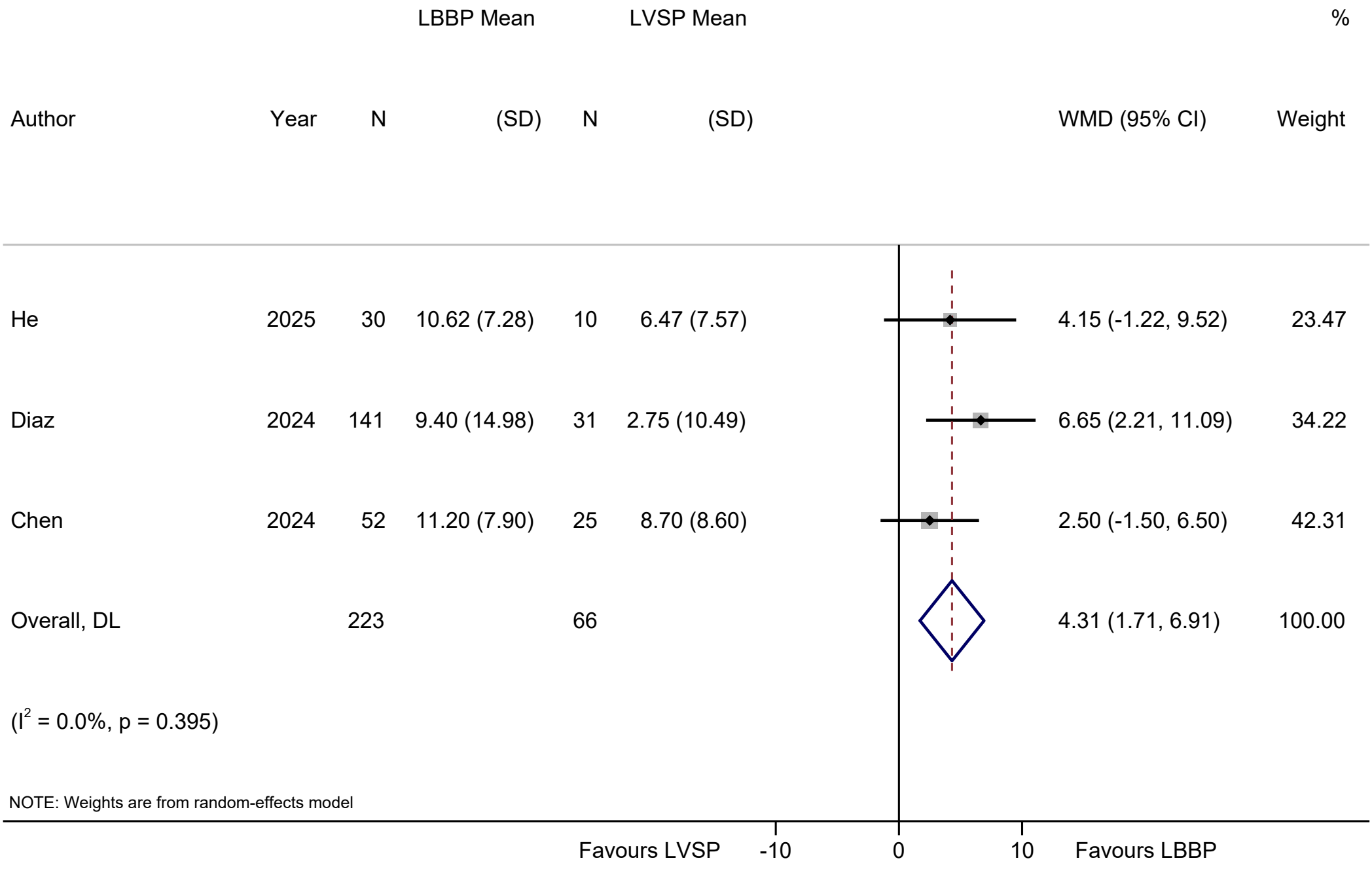
LBBP Mean
LVSP Mean
%
Author
Year
N
(SD)
N
(SD)
WMD (95% CI)
Weight
He
2025
30
10.62 (7.28)
10
6.47 (7.57)
4.15 (-1.22, 9.52)
23.47
Diaz
2024
141
9.40 (14.98)
31
2.75 (10.49)
6.65 (2.21, 11.09)
34.22
Chen
2024
52
11.20 (7.90)
25
8.70 (8.60)
2.50 (-1.50, 6.50)
42.31
Overall, DL
223
66
4.31 (1.71, 6.91)
100.00
(I2 = 0.0%, p = 0.395)
NOTE: Weights are from random-effects model
Favours LVSP
-10
0
10
Favours LBBP
