# Supplementary material for: Left bundle branch pacing is a superior pacing strategy compared to left ventricular septal pacing: insights from the pooled clinical evidence
Source: Front Physiol. 2026 Aug 24;17:1908837. doi: 10.3389/fphys.2026.1908837 (PMC13546746; doi:10.3389/fphys.2026.1908837)
Supplement: Supplementary file 3 [file DataSheet4.pdf]

PRISMA 2020 flow diagram for new systematic reviews which included searches of databases and registers only

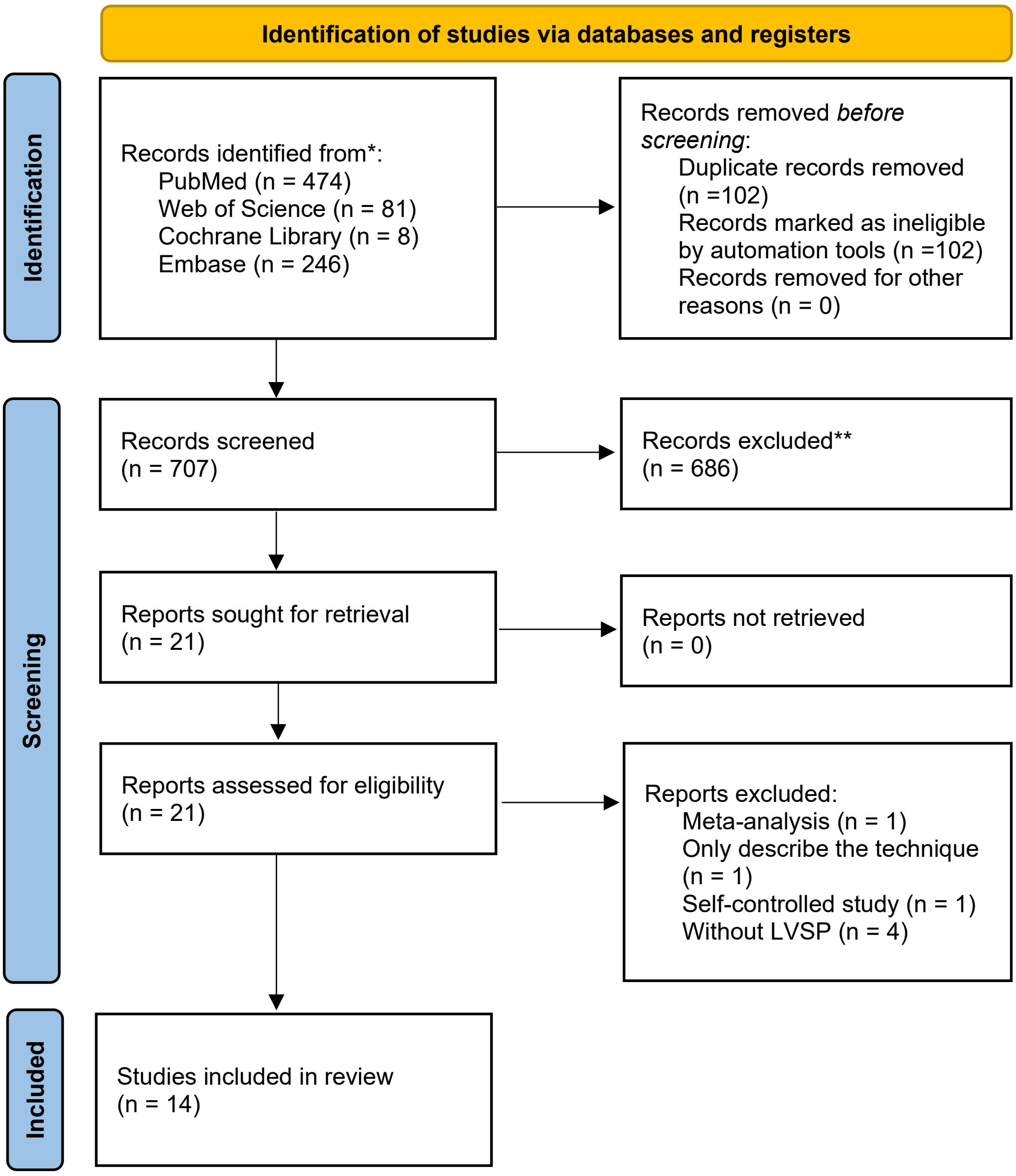

*Consider, if feasible to do so, reporting the number of records identified from each database or register searched (rather than the total number across all databases/registers).

**If automation tools were used, indicate how many records were excluded by a human and how many were excluded by automation tools.

From: Page MJ, McKenzie JE, Bossuyt PM, Boutron I, Hoffmann TC, Mulrow CD, et al. The PRISMA 2020 statement: an updated guideline for reporting systematic reviews. BMJ 2021;372:n71. doi: 10.1136/bmj.n71

For more information, visit: http://www.prisma-statement.org/
