# Supplementary material for: Left bundle branch pacing is a superior pacing strategy compared to left ventricular septal pacing: insights from the pooled clinical evidence
Source: Front Physiol. 2026 Aug 24;17:1908837. doi: 10.3389/fphys.2026.1908837 (PMC13546746; doi:10.3389/fphys.2026.1908837)
Supplement: Supplementary file 4 [file DataSheet1.docx]

The detail search strategy

Due to multiple novel pacing concepts involed in the eligible studies, the related medical subject headings (MESH) terms were unavalible. Therefore, keywords derived from the published articles, or alternative spellings were used for the search strategy. The detail search strategy with four online database was as follows:

**PubMed**

#1	"left bundle branch pacing" OR "LBBP"	**3,368**

#2	" left ventricular septal pacing " OR "LVSP"	**1956**

#3	#1 AND #2	**474**

**Web Of Science**

#1 "left bundle branch pacing" OR "LBBP" (Topic) and Preprint Citation Index (Exclude – Database) **732**

#2 left ventricular septal pacing" OR "LVSP" (Topic) and Preprint Citation Index (Exclude – Database) **1,683**

#3 #1 AND #2 and Preprint Citation Index (Exclude – Database)" **81**

**Cochrane Library**

#1	"left bundle branch pacing" OR "LBBP"	**78**

#2	" left ventricular septal pacing " OR "LVSP"	**19**

#3	#1 AND #2	**8**

**Embase database**

#1. ' left bundle branch pacing '/exp OR ' LBBP ' **2987**

#2. ' left ventricular septal pacing '/exp OR ' LVSP '	**1235**

#3. #1 AND #2 **246**

**The excluded reports before determining the finial eligible studies:**

1. Meta-analysis (n = 1)

- Putra ICS, Pranata R, Iqbal M, Karwiky G, Achmad C. Efficacy and safety of left bundle branch pacing compared with left ventricular septal pacing: A systematic review and meta-analysis. Heart Rhythm O2. 2025;6(9):1335-1355. Published 2025 Aug 13. doi:10.1016/j.hroo.2025.06.006

2. Only describe the technique (n = 1)

- Ponnusamy SS, Pillai A, Burkhoff D, et al. Hierarchical physiologic pacing to enhance clinical outcomes in conduction system pacing. *Heart Rhythm*. 2026;23(1):114-122. doi:10.1016/j.hrthm.2025.04.035

3. Self-controlled study (n = 1)

- Estévez Paniagua Á, Briongos-Figuero S, Tapia Martínez M, et al. Mid- to long-term mechanical performance of left bundle branch area pacing: a comprehensive echocardiographic comparison of capture modalities. Europace. 2025;27(11):euaf271. doi:10.1093/europace/euaf271

4. Without LVSP (n = 4)

- Liu X, Li W, Zhou X, Huang H, Wang L, Wu M. Clinical Outcomes of Left Bundle Branch Area Pacing in Comparison with Right Ventricular Septal Pacing in Patients with High Ventricular Pacing Ratio ≥40. *Int J Gen Med*. 2022;15:4175-4185. Published 2022 Apr 19. doi:10.2147/IJGM.S360522
- Mei Y, Han R, Cheng L, et al. Assessment of Cardiac Function and Ventricular Mechanical Synchronization in Left Bundle Branch Area Pacing by Speckle Tracking and Three-Dimensional Echocardiography. *Am J Cardiol*. 2023;187:1-9. doi:10.1016/j.amjcard.2022.10.012
- Sato T, Togashi I, Ikewaki H, et al. Diverse QRS morphology reflecting variations in lead placement for left bundle branch area pacing. *Europace*. 2023;25(9):euad241. doi:10.1093/europace/euad241
- Ramalingam V, Ponnusamy S, Abdulkader RS, et al. Is left bundle branch pacing (LBBP) associated with better depolarization and repolarization kinetics than right ventricular mid septal pacing (RVSP)? - Comparison of frontal QRS -T angle in patients with LBBP, RVSP and normal ventricular conduction. *Indian Pacing Electrophysiol J*. 2024;24(2):75-83. doi:10.1016/j.ipej.2023.12.004
