# Supplementary material for: Left bundle branch pacing is a superior pacing strategy compared to left ventricular septal pacing: insights from the pooled clinical evidence
Source: Front Physiol. 2026 Aug 24;17:1908837. doi: 10.3389/fphys.2026.1908837 (PMC13546746; doi:10.3389/fphys.2026.1908837)
Supplement: Supplementary file 5 [file Table1.docx]

**Supplementary Table 1.** Quality assessment of eligible studies according to the Newcastle-Ottawa Quality Assessment Scale (NOS)

| First author | Year | Selection |  |  |  | Comparability | Outcome |  |  | Total stars |
| --- | --- | --- | --- | --- | --- | --- | --- | --- | --- | --- |
|  |  | Representativeness of the exposed cohort | Selection of the nonexposed cohort | Ascertainment of exposure | Demonstration that outcome of interest was not present at start of study | Comparability of cohorts on the basis  of the design or analysis | Assessment of outcome | Was follow-up long enough for outcomes to occur | Adequacy of follow-up of cohorts |  |
| Briongos | 2025 | ***** | ***** | ***** | ***** | ****** | ***** | ***** | ***** | **9** |
| Bertini | 2025 | ***** | ***** | ***** | ***** | ****** | ***** |  | ***** | **8** |
| He | 2025 | ***** | ***** | ***** | ***** | ****** | ***** | ***** | ***** | **9** |
| Cheng | 2024 | ***** | ***** | ***** | ***** | ****** | ***** | ***** | ***** | **9** |
| Zhu | 2024 | ***** | ***** | ***** | ***** | ****** | ***** | ***** | ***** | **9** |
| Rijks | 2024 | ***** | ***** | ***** | ***** | ****** | ***** |  | ***** | **8** |
| Diaz | 2024 | ***** | ***** | ***** | ***** | ****** | ***** | ***** | ***** | **9** |
| Chen | 2024 | ***** | ***** | ***** | ***** | ****** | ***** | ***** | ***** | **9** |
| Zhang-2 | 2023 | ***** | ***** | ***** | ***** | ****** | ***** | ***** | ***** | **9** |
| Peng | 2023 | ***** | ***** | ***** | ***** | ****** | ***** |  | ***** | **8** |
| Kato | 2023 | ***** | ***** | ***** | ***** | ****** | ***** |  | ***** | **8** |
| Shimeno | 2022 | ***** | ***** | ***** | ***** | ****** | ***** |  | ***** | **8** |
| Zhou | 2022 | ***** | ***** | ***** | ***** | ****** | ***** | ***** | ***** | **9** |
| Zhang-1 | 2021 | ***** | ***** | ***** | ***** | ****** | ***** | ***** | ***** | **9** |
